# High-performance solid-state supercapacitors based on graphene-ZnO hybrid nanocomposites

**DOI:** 10.1186/1556-276X-8-473

**Published:** 2013-11-12

**Authors:** Zijiong Li, Zhihua Zhou, Gaoqian Yun, Kai Shi, Xiaowei Lv, Baocheng Yang

**Affiliations:** 1 Institute of Nano Functional Materials, Huanghe University of Science & Technology, Zhengzhou 450006, People's Republic of China; 2 School of Physics & Electronic Engineering, Zhengzhou University of Light Industry, Zhengzhou 450002, People's Republic of China; 3 School of Microelectronics and Solid-State Electronics, University of Electronic Science and Technology of China, Chengdu 610054, People's Republic of China

**Keywords:** Zinc oxide nanorods, Graphene nanosheets, Solid-state supercapacitor

## Abstract

In this paper, we report a facile low-cost synthesis of the graphene-ZnO hybrid nanocomposites for solid-state supercapacitors. Structural analysis revealed a homogeneous distribution of ZnO nanorods that are inserted in graphene nanosheets, forming a sandwiched architecture. The material exhibited a high specific capacitance of 156 F g^−1^ at a scan rate of 5 mV.s^−1^. The fabricated solid-state supercapacitor device using these graphene-ZnO hybrid nanocomposites exhibits good supercapacitive performance and long-term cycle stability. The improved supercapacitance property of these materials could be ascribed to the increased conductivity of ZnO and better utilization of graphene. These results demonstrate the potential of the graphene-ZnO hybrid nanocomposites as an electrode in high-performance supercapacitors.

## Background

As a new class of energy storage device, supercapacitors, also known as electrochemical capacitors, has received considerable attention that can be used in hybrid electric vehicles, memory backup, and other emergency power supply devices due to their higher power density, superior cycle lifetime, and low maintenance cost. However, the energy density of supercapacitors is lower than batteries [1-6]. It is highly desirable to increase the energy density of supercapacitors to approach that of batteries, which could enable their use as primary power sources. Supercapacitors store electrical energy by two mechanisms [7,8]: electrochemical double-layer capacitance (EDLC) and pseudocapacitance. In EDLC, the capacitance comes from the charge accumulated at the electrode-electrolyte interface. Carbon-based materials are widely used in EDLC electrode due to their high surface area and excellent electric conductivity. Compared to EDLCs, pseudocapacitors can provide much higher capacitance and energy density through Faradic reaction [6,7]. Transition metal oxides and conducting polymers are the promising candidates because they can provide high energy density for pseudocapacitors. It has been found that carbon materials which combine with pseudocapacitive electrode materials can improve the capacitance of supercapacitors [8-10].

Graphene (Gr) is an atom-thick, two-dimensional (2D) material composed of a monolayer hexagonal *sp*^2^-hybridized carbon. Gr with the maximum surface area of 2,630 m^2^ g^−1^ and high intrinsic electrical conductivity is believed to be one of the most promising electrode materials for supercapacitors [11-14]. However, in practical applications, Gr nanosheets usually suffer from agglomeration or restacking due to strong van der Waals interactions [15-17], which leads to the loss of surface area and capacitance. Metal/metal oxide or metal hydroxide nanoparticles are currently introduced into the interlayer of Gr nanosheets to prevent agglomeration [18-21]. Transition metal oxides [22-25], which can contribute to pseudocapacitance such as RuO_2_, have been recognized as the best electrode materials for supercapacitors. However, their expensive nature and high toxicity severely limit their practical application in a large scale. Therefore, the development of low-cost and high-abundance metal oxide as an alternative is highly desirable [26-29]. ZnO is considered to be a promising material for supercapacitors due to its high specific energy density, low cost, non-toxicity, eco-friendliness, and abundant availability.

Very recently, Kim et al. [30] and Pan et al. [31] reported on reduced graphene oxide-ZnO nanocomposites for supercapacitor electrodes by microwave-assisted method, which exhibited a specific capacitance of 109 F g^−1^ at a scan rate of 2 mV s^−1^ and 146 F g^−1^ at a scan rate of 2 mV s^−1^, respectively. But only approximately 30 F g^−1^ at a scan rate of 100 mV s^−1^. A sandwiched nanoarchitecture of reduced graphene oxide/ZnO/deducted graphene oxide is fabricated by Huang et al. [32] using chemical vapor deposition method, which exhibited a specific capacitance of 51.6 F g^−1^ at a scan rate of 10 mV s^−1^. Additionally, graphene-ZnO nanocomposites synthesized by other method such as ultrasonic spray pyrolysis method and their electrochemical performance were reported [33,34]. However, these materials were limited by a low specific capacitance and poor stability at higher scan rate or high current densities. An effective regulation of graphene-ZnO hybrid for high performance of supercapacitors is still challenging. On the other hand, the investigation of solid-state supercapacitors based on graphene-ZnO hybrid is very limited.

In this report, a simple and facile synthesis route is developed to prepare graphene-ZnO hybrid as an electrode material for supercapacitors using one-step hydrothermal technique. Initially, graphene oxide (GO) was synthesized using the well-known modified Hummer's method. ZnO nanorods are inserted between the graphene nanosheets layer-by-layer rather than simply decorated on the surface of graphene during GO hydrothermal reduction process. This strategy provides a novel method for the preparation of highly active materials (ZnO nanorods) directly grown on Gr surface that avoids the restacking of Gr sheets, which show high specific capacitance even at higher scan rate and excellent long-term cycle stability applied in a all solid-state supercapacitor device. Such high electrochemical properties provide important prospects for graphene-ZnO hybrid to be widely used as electrode material in supercapacitor.

## Methods

### Materials

Graphite powder was purchased from Sigma Aldrich (St. Louis, MO, USA). All other reagents were commercially available and analytic grade and were used directly without any purification. Double-distilled water was used throughout the experiments.

### Synthesis of graphene oxide

Graphite oxide was prepared from natural graphite powder through a modified Hummers method [35]. One gram of graphite powder, 1.1 g sodium nitrate, and 46 ml sulfuric acid were mixed and stirred for 10 min. Then, 3.0 g potassium permanganate was added slowly and temperature maintained below 20°C. DI water was added slowly and the temperature was raised to 90°C. The solution turned bright yellow when 3.0 ml of hydrogen peroxide (30%) was added. The mixture was filtered while warm and washed with warm DI water. Then GO was subjected to dialysis to completely remove metal ions and acids. Finally, the product was dried in air at room temperature.

### Synthesis of ZnO nanorods

Pure ZnO nanorods were synthesized by hydrothermal method. In a typical experiment, 100 mg of Zn(NO_3_)_2_ was first dispersed into 30 ml deionized water. Then, 15 μl of hydrazine hydrate was added drop by drop under stirring, followed by ultrasonication for 30 min. Then the solution was transferred to a 50 ml of Teflon-lined autoclave and heated at 160°C for 12 h. Finally, the ZnO nanostructures were collected after washing and centrifugation.

### Synthesis of the graphene-ZnO hybrid nanostructure

As-synthesized GO (50 mg) was dispersed in 100 ml double-distilled water; the dispersion was brown in color. The dispersed GO was exfoliated, using sonication for 1 h, and then 20 mg Zn(NO_3_)_2_ and 10 μl hydrazine hydrate were added into the abovementioned solution under ultrasonication. After hydrothermal reaction at 160°C for 12 h, the graphene-ZnO nanocomposites were synthesized and collected through washing, centrifugation, and drying.

### Characterizations

The microstructure morphologies and crystal structures of the as-synthesized pure ZnO, pristine graphene, and graphene-ZnO nanocomposites were characterized using field-emission scanning electron microscope (FESEM, Quanta 250 FEG; FEI, Hillsboro, OR, USA), X-ray diffraction (XRD, D8 ADVANCE, Bruker, Billerica, MA, USA) with Cu-Kα radiation (λ = 0.154178 nm), transmission electron microscopy (TEM) (JEM2010-HR, JEOL, Akishima, Tokyo, Japan), and laser micro-Raman spectrometry (Renishaw inVia, Gloucestershire, UK). Energy dispersive spectrometer (EDS) mapping analysis was used to analyze the element distribution of the as-synthesized nanocomposites. Inductively coupled plasma atomic emission spectroscopy (ICP, SPECTRO, Birmingham, UK) was used to analyze the loading of ZnO on graphene. The electrochemical measurements were carried out on a CHI 660D electrochemical workstation (Chenhua, Shanghai, China) at room temperature.

### Preparation of electrodes and electrochemical characterization

The working electrode was prepared as follows: approximately 10 mg of as-synthesized material was first mixed with polytetrafluoroethylene (60 wt.% water suspension; Sigma-Aldrich, St. Louis, MO, USA) in a ratio of 100:1 by weight and then dispersed in ethanol. The suspension was drop-dried into a 1 cm × 1 cm Ni foam (2-mm thick) at 80°C. The sample loaded foam was compressed before measurement.

The electrochemical measurements including cyclic voltammograms (CVs), galvanostatic charge/discharge, and electrochemical impedance spectroscopy were performed in a three-electrode setup: a Ni foam coated with the active materials serving as the working electrode, a platinum foil electrode, and a saturated calomel electrode (SCE) serving as the counter and reference electrodes, respectively.

### Fabrication of solid-state supercapacitors

The device was assembled by two pieces of graphene-ZnO electrodes with a separator (Whatman 8-μm filter paper) sandwiched in between and polyvinyl alcohol (PVA)-gelled as a solid electrolyte. The PVA-gel electrolyte was made by following method. 600 mg PVA was mixed with 5 ml Milli-Q water (Millipore Corp., Billerica, MA, USA). The mixture was heated at 80°C under stirring for 30 min and then cooled naturally. Then approximately 10 ml of 0.5 M NaNO_3_ was added to the mixture and stirred for 30 min. The graphene-ZnO hybrid materials were collected on a Teflon membrane (0.2-m pore size) by vacuum filtration and then pressed onto the carbon-coated Al current collector. The graphene-ZnO electrodes and a separator were sandwiched together in a stainless steel cell for the fully assembled two-electrode cell device.

## Results and discussion

**Figure 1:**
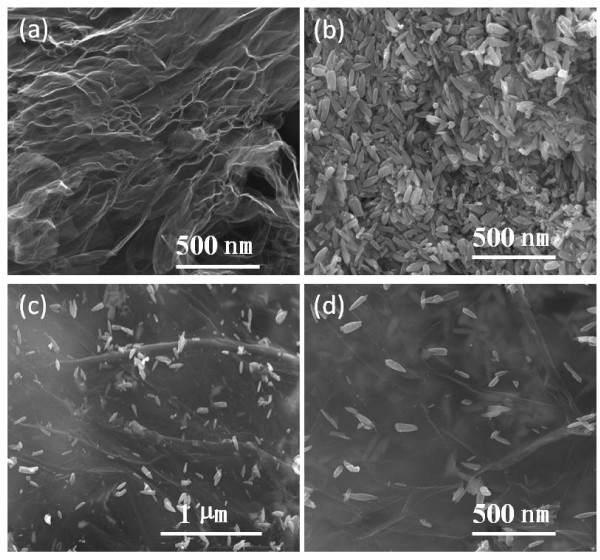
**SEM images.** GO **(a)**, ZnO **(b)**, low and high magnification of graphene-ZnO hybrid nanostructure **(c, d)**.

Figure 1 shows the typical images of pristine GO, ZnO, and the as-synthesized graphene-ZnO nanocomposites. Figure 1a presents SEM images of the GO film, showing a stack of layered laminas composed of complex fold and pockets of void space. It is conspicuous to observe the edges of individual sheets, including the crumpled and continuous areas. The ZnO nanorods with smooth surface and high crystallinity can be observed from Figure 1b. The diameters of ZnO nanorods are typically in the range of approximately 20 to 30 nm. After ZnO was inserted in GO sheets by hydrothermal method, typical SEM images were taken and are shown in Figure 1c,d. It is found that the ZnO nanorods are dispersed uniformly on the surface of Gr. The ZnO nanorods were sandwiched in between Gr layers so that Gr sheets are loosely stacked into continuous films without apparent stacking order.

**Figure 2:**
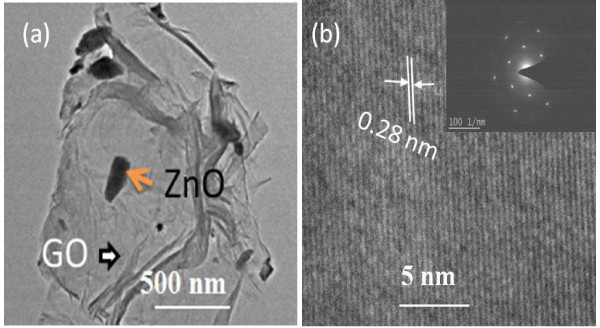
**TEM image (a) and HRTEM image (b) of graphene-ZnO nanocomposites.** Inset of **(b)** is the corresponding SAED pattern.

The TEM image (Figure 2a) identifies that the ZnO nanorods with an average diameter of approximately 20 nm are dispersed into the Gr layers. The uniform distribution of ZnO nanorods among the Gr is due to the *in situ* hydrothermal reduction on the surface of Gr. The high-resolution TEM (HRTEM) and the selected-area electron diffraction (SAED) pattern of the graphene-ZnO hybrid nanostructure (Figure 2b) confirmed the hexagonal wurtzite phase of ZnO nanorods.

**Figure 3:**
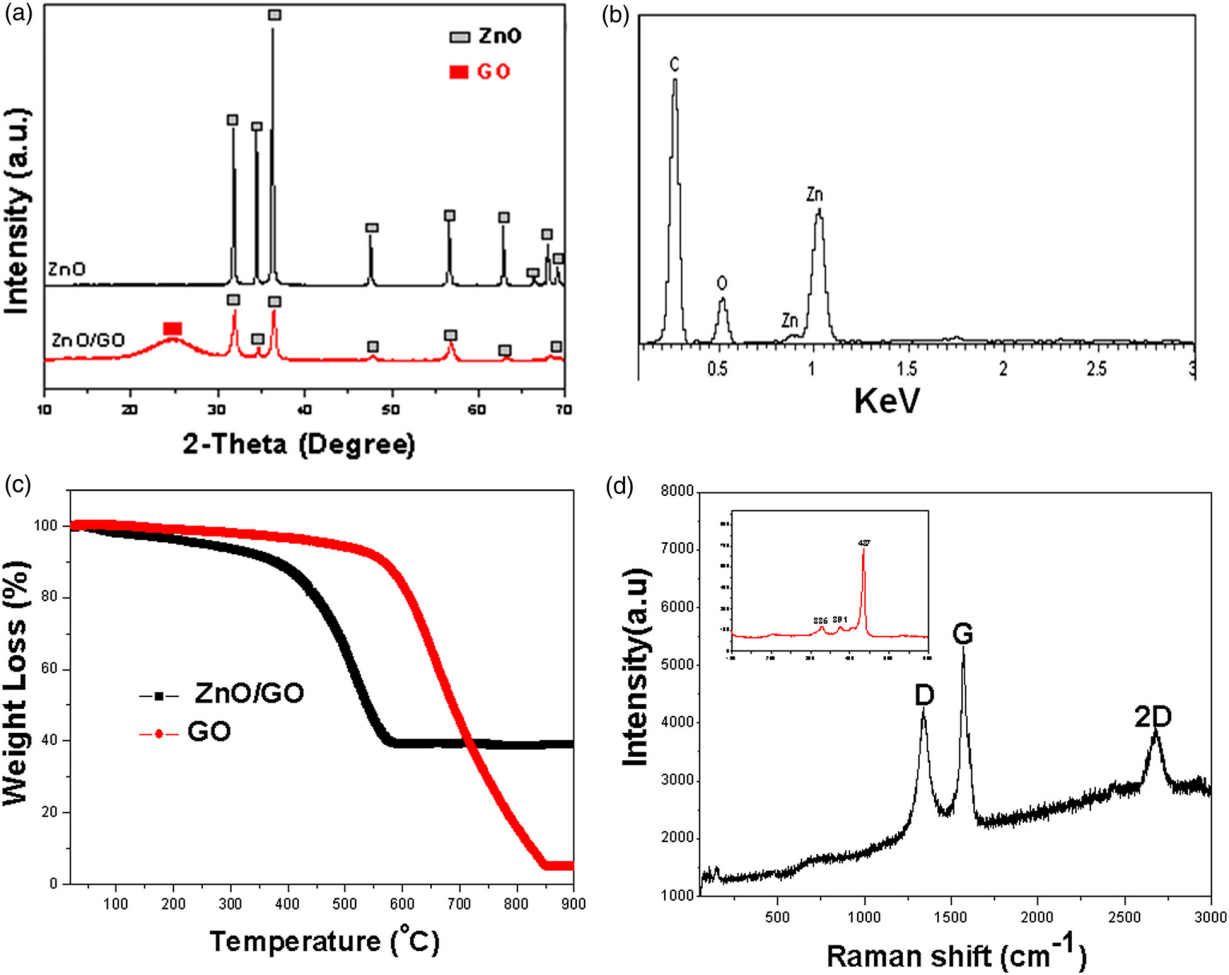
**Characterization of ZnO, graphene-ZnO, graphene-ZnO hybrid nanostructures. (a)** XRD patterns of ZnO and graphene-ZnO. **(b)** EDS image of the graphene-ZnO hybrid nanostructure. **(c)** TGA curves of GO and graphene-ZnO sample, heating rate 10°C min^−1^. **(d)** Raman spectra of graphene-ZnO hybrid nanostructure.

Figure 3a shows the typical XRD patterns of ZnO and the as-synthesized graphene-ZnO hybrid nanostructure. It is found that the XRD pattern of ZnO consists of five diffraction peaks at 32.6°, 35°, 36.8°, 47.8°, 56.5°, 62.5°, and 67.6°, corresponding to the (100), (002), (101), (102), (110), (103), and (112) planes of the hexagonal wurtzite ZnO phase (JCPDS 65–3411), respectively. From the XRD pattern of the graphene-ZnO hybrid nanostructure, a strong and broad peak appeared at a 2*θ* value of 25°, which corresponded to the (002) plane of Gr . No other peaks of GO observed indicate that GO is completely reduced to a Gr sheet. Other peaks observed in the XRD pattern matched the hexagonal wurtzite ZnO, indicating a well hybrid. The EDS spectrum of the graphene-ZnO hybrid nanostructure is shown in Figure 3b. The spectrum clearly showed the presence of carbon (C), zinc (Zn), and oxygen (O) elements in the graphene-ZnO hybrid nanostructure. The Zn and O elements originated from the ZnO nanorods, and the C was contributed by the Gr nanosheets. Thermogravimetric analysis (TGA) of Sn-Gr composite was performed to find out metal oxide content in the sample. Figure 3c shows the TGA profiles of GO and graphene-ZnO hybrid nanostructure measured in air conditions. After the product had been calcined at 900°C in air, the residue of GO is approximately 5 wt.%, while the graphene-ZnO hybrid sample is approximately 38.5 wt.%. Therefore, the ZnO content in the graphene-ZnO sample was determined to be about 33.5 wt.%. In addition, the lower thermal stability of the graphene-ZnO compared to the pristine GO may be due to the catalytic decomposition of ZnO since carbon has been reported to catalytically decompose oxides. To further confirm the formation of the samples, Raman detection was performed. Figure 3d shows the Raman spectra of graphene-ZnO hybrid nanostructure. A very intense Raman band can be seen at 1,354 and 1,596 cm^−1^, which corresponded to the well-documented D and G bands, respectively. The D band is a common feature for *sp*^3^ defects or disorder in carbon, and the G band provides useful information on in-plane vibrations of *sp*^2^-bonded carbon atoms in a 2D hexagonal lattice. The 2D band appeared in the sample, indicating the conversion of GO into Gr sheets. Further observation showed that three vibrational peaks at 323, 437, and 487 cm^−1^ were also observed (inset in Figure 3d), which correspond to the to the optical phonon *E*_2_ mode of wurtzite hexagonal phase of ZnO.

**Figure 4:**
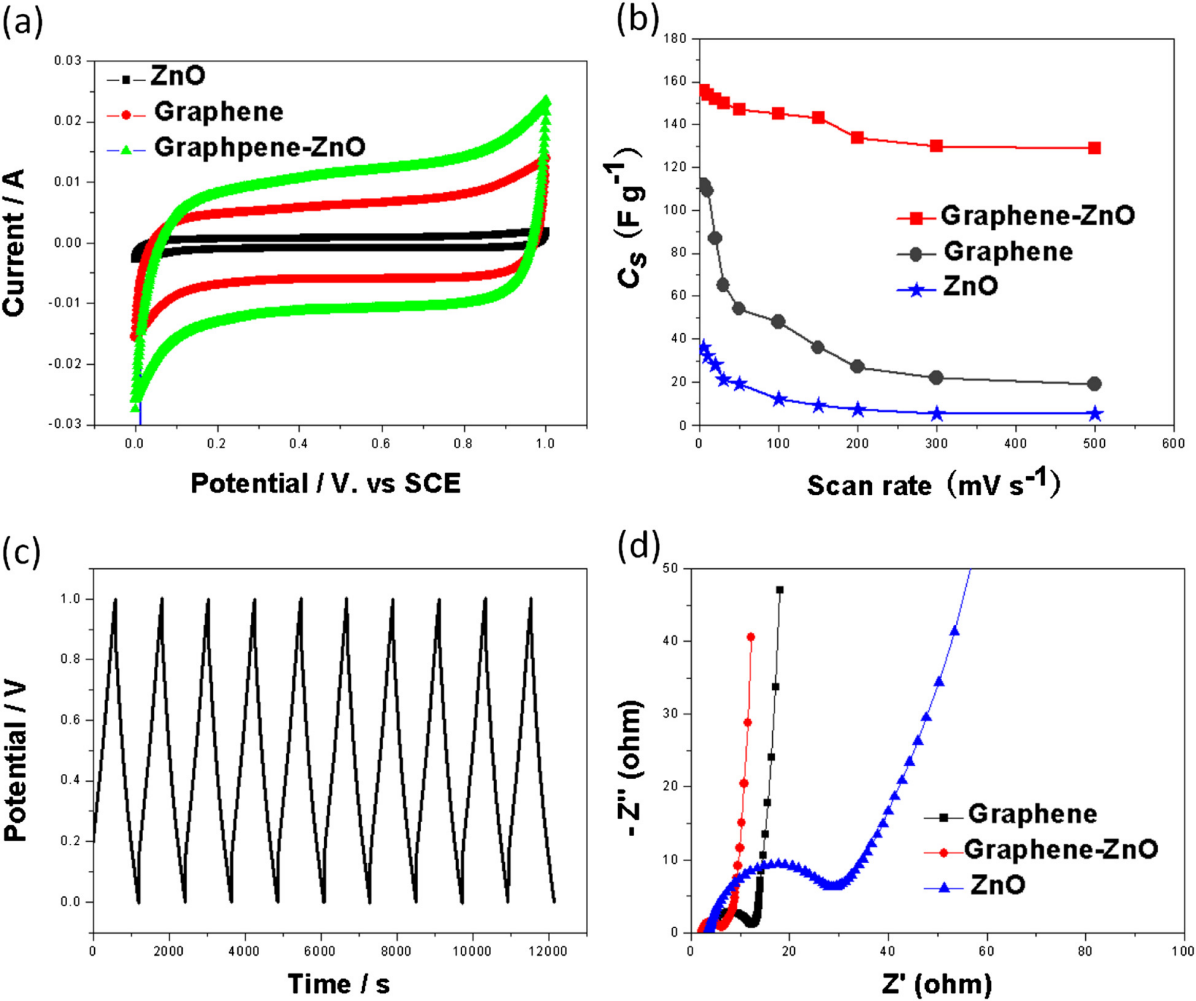
**CV curves, specific capacitance, galvanostatic charge–discharge curve, and Nyquist plots of electrodes. (a)** CV curves of the as-prepared ZnO, graphene and the graphene-ZnO hybrid electrode at a scan rate of 5 mV s^−1^ in 0.5 M Na_2_SO_4_ electrolyte solution. **(b)** Specific capacitance of ZnO, pristine graphene, and the graphene-ZnO hybrid electrode at different scan rates calculated from CV curves. **(c)** Galvanostatic charge–discharge curve of the graphene-ZnO hybrid electrode at a constant current density of 2.0 mA cm^−2^. **(d)** Nyquist plots for ZnO, pristine graphene, and the graphene-ZnO hybrid electrode.

To study the electrochemical performance of the graphene-ZnO hybrid nanostructure, electrochemical measurements were conducted in a three-electrode electrochemical cell with a Pt wire as counter electrode and a SCE as reference electrode in 0.5 M Na_2_SO_4_ solution. In order to illustrate the advantage of the graphene-ZnO hybrid nanostructure, Figure 4a compares the cyclic voltammetry (CV) curves of pristine Gr sheets, ZnO nanorods, and graphene-ZnO hybrid nanostructure at 5 mV s^−1^. It can be seen that all these curves exhibit nearly rectangular shape, indicating ideal supercapacitive behavior. In comparison to the ZnO nanorods and pristine Gr electrodes, the graphene-ZnO hybrid nanostructure electrode showed a higher integrated area, which reveals the superior electrochemical performance of the graphene-ZnO hybrid electrode. The specific capacitance (*C*_s_) values were calculated from the CV curves using the following equation [36]:

(1) $$C_{s}=\frac{\int I\left(u\right)dt}{m\times v\times \Delta V}$$

where *I* is the oxidation or reduction current, d*t* is time differential, *m* indicates the mass of the active electrode material, and *∆V* indicates the voltage range of one sweep segment. According to Equation 1, the calculated *C*_s_ values of ZnO nanorods, pristine Gr sheets, and the graphene-ZnO hybrid electrode are 36, 112, and 156 F g^−1^, respectively, at a scan rate of 5 mV s^−1^. The specific capacitance of the graphene-ZnO hybrid electrode was much higher than that of the ZnO nanorods and pristine Gr sheets. Moreover, this value is higher than that of previously reported. To obtain a more detailed information on the capacitance performance of the as-prepared graphene-ZnO hybrid nanostructure, the CV curves with various scan rates were studied. Figure 4b summed the *C*_s_ of ZnO, pristine Gr, and graphene-ZnO hybrid electrodes at various scan rates. It can be seen that the specific capacitance decreased with an increase in the scan rate from 5 to 500 mV s^−1^. The reason may be that insufficient time available for ion diffusion and adsorption inside the smallest pores within a large particle at high scan rates [37]. Moreover, the *C*_s_ of the graphene-ZnO hybrid electrode was much higher than that of a ZnO and pristine Gr electrodes for all the scan rates tested. Figure 4c shows galvanostatic charge–discharge measurements of the graphene-ZnO hybrid electrode at a constant current density of 2.0 mA cm^−2^. It can be seen that the curves were linear and exhibited a typical triangular shape even charging/discharging for 12,000 s, which indicated good electrochemical capacitive characteristics. The enhanced electrochemical performance of the graphene-ZnO hybrid can be attributed to the sandwiched structure. Here, the graphene in the hybrid electrode provides better electronic conductivity and excellent interfacial contact between ZnO and graphene, which results in the fast transportation of electrons throughout the entire electrode matrix [38]. Moreover, it is evident that when the ZnO size is reduced to nanometer dimensions, the surface area and electroactive sites increase, which effectively reduces the diffusion length of the Na^+^ ion in the electrode matrix [39,40].

**Figure 5:**
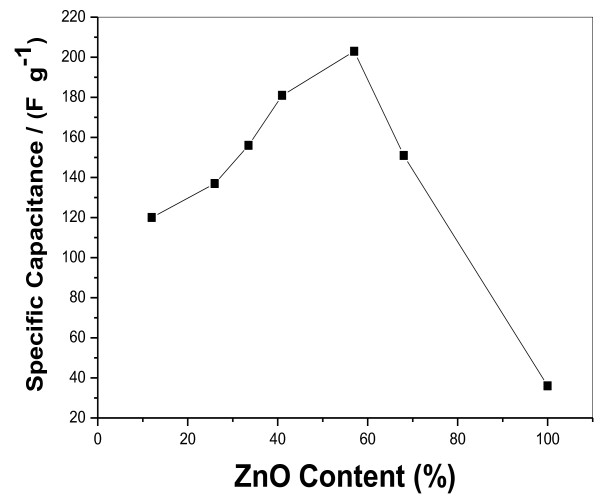
Effects of ZnO amount on electrochemical properties.

Electrochemical impedance spectroscopy (EIS) was conducted to understand the conductivity, mechanistic analysis of interfacial processes and structure, and charge transport in the material/electrolyte interface of these electrodes. Figure 4d shows the Nyquist plots for the ZnO, pristine Gr, and graphene-ZnO hybrid electrodes. All these plots display a semicircle in the high-frequency region and a straight line in the low-frequency region. The straight line in the low-frequency range is called the Warburg resistance, which is caused by the frequency dependence of ion diffusion/transport from the electrolyte to the electrode surfaces [41]. The arc for the very high-frequency range corresponded to the charge transfer limiting process and was ascribed to the double-layer capacitance in parallel with the charge transfer resistance (Rct) at the contact interface between the electrode and electrolyte solution [42]. The Rct can be directly measured from the Nyquist plots as the semicircular arc diameter. The Rct for the graphene-ZnO hybrid electrode is 3.5 Ω, which is substantially smaller than those of pristine ZnO (26.4 Ω) and Gr (8.2 Ω) electrodes, indicating the better conductivity of the graphene-ZnO hybrid electrode. It indicated the incorporation of ZnO nanorods into the graphene nanosheets, resulting in an improved charge transfer performance for the electrode. Figure 5 showed the effects of ZnO amount on electrochemical properties. It can be seen that increasing the ZnO content can improve the electrochemical properties of graphene-ZnO hybrid. However, the electrochemical properties of graphene-ZnO hybrid decreased when the ZnO content is excess 60%. The reason is due to the poor conductivity of ZnO.

**Figure 6:**
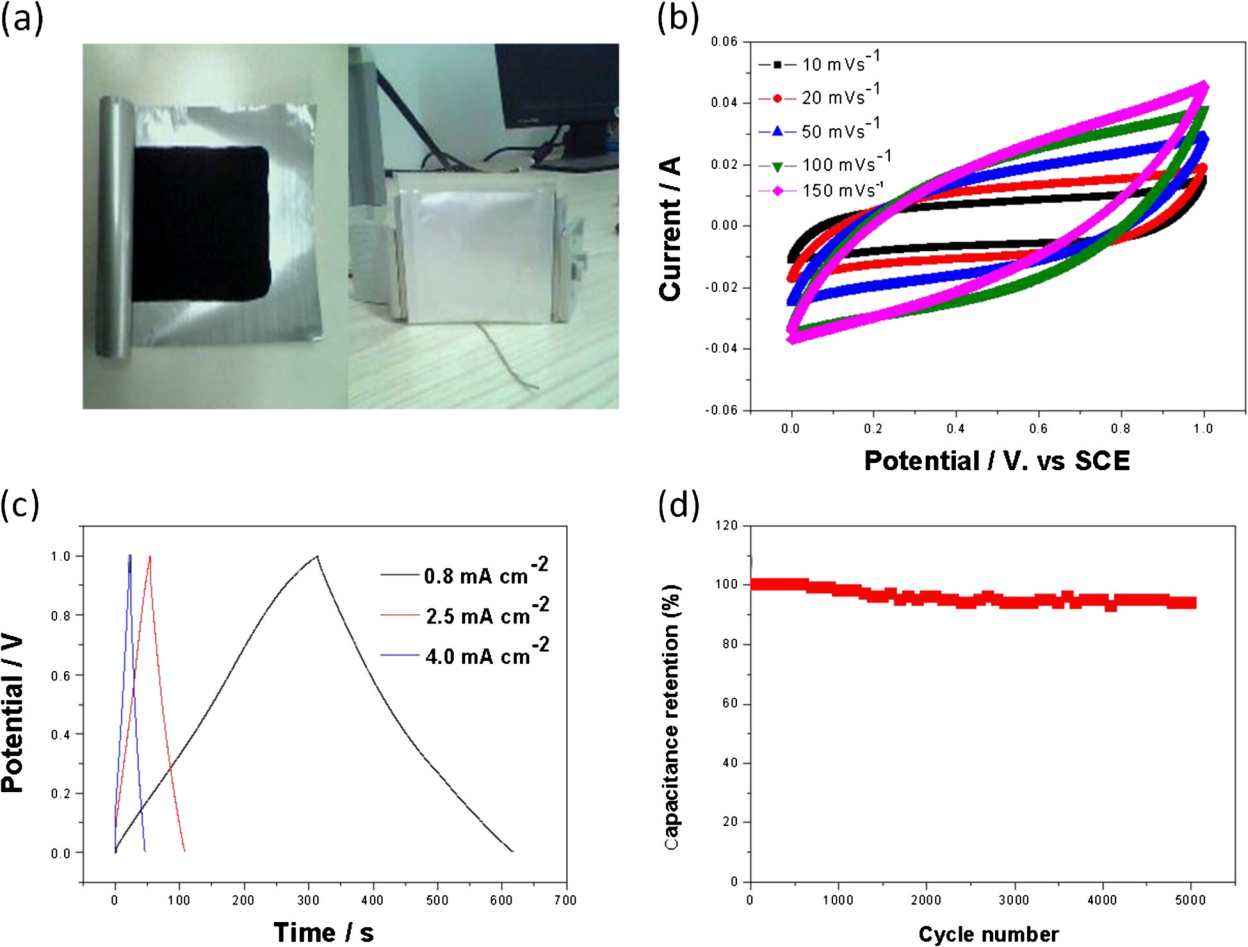
**Supercapacitance properties of graphene-ZnO hybrid in all-solid supercapacitors. (a)** Fabricated solid-state supercapacitor device-based graphene-ZnO hybrid electrode. **(b)** CV curves of the graphene-ZnO hybrid electrode at different scan rates from 10 to 150 mV s^−1^. **(c)** Galvanostatic charge–discharge curves of the graphene-ZnO hybrid electrode at different current densities. **(d)** Variation of the specific capacitance of the graphene-ZnO hybrid electrode as a function of cycle number.

To test their feasibility for application as an energy storage device, solid-state symmetrical supercapacitors based on graphene-ZnO hybrid were fabricated by sandwiching H_2_SO_4_-PVA-based solid-state electrolyte between two pieces of graphene-ZnO electrodes (Figure 6a). CV curves of the solid-state supercapacitor device measured at various scan rates are collected in Figure 6b. All the CV curves exhibit a rectangular-like shape, which reveals the ideal capacitive behavior and fast charge–discharge behavior. Figure 6c shows the galvanostatic charge–discharge curves of the solid-state supercapacitor device collected at different current densities. The discharge curves of this device are relatively symmetrical with its corresponding charge counterparts, confirming the good capacitive behavior and fast charge–discharge behavior of the fabricated supercapacitor device. The specific capacitance for the electrodes can be obtained from charge–discharge data according to Equation 2

(2) $$C=\frac{I\times \Delta t}{m\times \Delta V}$$

where *C* (F g^−1^) is the specific capacitance, *I* (A) is the constant discharging current, ∆*t* (s) is the discharging time, ∆*V* (V) is the potential window, and *m* (g) is the mass loading of the active material in the working. The specific capacitances of the graphene-ZnO hybrid electrode are 196, 115, and 102 F g^−1^ at the current densities of 0.8, 2.5, and 4.0 mA cm^−2^, respectively. Additionally, the specific capacitance values decreased with increasing current density. However, the present values are higher than the previously reported even at high current density. The average energy density (*E*) and power density (*P*) were derived from the CV curves at different scan rates using the following equations [43]:

(3) $$E=\frac{0.5\times C{\left(\mathrm{\Delta V}\right)}^{2}}{3.6}$$

(4) $$P=\frac{E\times 3,600}{\mathrm{\Delta t}}$$

where *E* is the average energy density of the electrode (W h kg^−1^), *P* is the average power density (W kg^−1^), *C* is the specific capacitance of the active material (F g^−1^), ∆*V* is the voltage range of one sweep segment, and ∆*t* (s) is the time for a sweep segment. The calculated average energy density and power density of the graphene-ZnO hybrid electrode were approximately 21.7 W h kg^−1^ and 2.6 kW kg^−1^, respectively, at a scan rate of 5 mV s^−1^.

The long cycle life of the supercapacitors is an important parameter for their practical application. The cycle stability of the graphene-ZnO hybrid electrode was further evaluated by repeating the CV measurements between 0 and 1.0 V at a scan rate of 100 mV s^−1^ for 5,000 cycles. Figure 6d shows the capacitance retention ratio as a function of cycle number. The capacitance of graphene-ZnO hybrid electrode retained 94% of its initial capacitor after 5,000 cycles (Figure 6d), which demonstrates excellent electrochemical stability. From these results, we concluded that the graphene-ZnO hybrid electrode materials showed a higher specific capacitance, significantly improved energy density, and excellent cycling performance.

The better electrochemical performance of the as-prepared graphene-ZnO electrode can be attributed to the following aspects: On the one hand, Gr sheets in the hybrid structure can act as a conducting agent, which greatly improves the electrical conductivity of the hybrid structure. On the other hand, the small size of the ZnO nanorods uniformly dispersed between the Gr sheets can effectively prevent the agglomeration and restacking of the Gr nanosheets, resulting in an EDLC for the overall specific capacitance. At the same time, Gr nanosheet with a large surface area in the hybrid structure not only provided double-layer capacitance to the overall energy storage but also effectively inhibited the aggregation of ZnO nanorods, resulting in fast electron transfer throughout the entire electrode matrix as well as an overall improvement in the electrochemical performance. Moreover, the nanometer-sized smaller ZnO rods facilitate faster charge–discharge rates, because the faradaic reaction replaced the diffusion-controlled Na^+^ ion intercalation process which usually occurs at the ZnO surface [44]. Therefore, the supercapacitive performance of graphene-ZnO hybrid based supercapacitor is significant improved.

## Conclusions

In summary, the graphene-ZnO hybrid nanostructure as an electrode material for solid-state supercapacitors was successfully synthesized using one-step hydrothermal method. The surface morphology, microstructure, composition, and capacitive behaviors of the as-prepared materials were well investigated. SEM and TEM images revealed the uniform distribution of ZnO nanorods on the Gr sheet substrate. In comparison with the specific capacitance of ZnO and pristine Gr electrode, the specific capacitance of graphene-ZnO hybrid electrode (156 F g^−1^ at a scan rate of 5 mV s^−1^) is significantly improved. Moreover, the material exhibited excellent electrochemical stability. The improved supercapacitance performance of the graphene-ZnO hybrid was mainly attributed to the pseudocapacitance of the ZnO phase and the intrinsic double-layer capacitance of the Gr sheets. The low price, abundant resources, and environmental friendliness of ZnO may render their nanocomposites a promising candidate for practical applications.

## Competing interests

The authors declare that they have no competing interests.

## Authors’ contributions

ZL carried out the experiment and drafted the manuscript. ZZ and XL performed the statistical analysis. GY and KS conceived of the study. BY participated in its design and coordination. All authors read and approved the final manuscript.
